# Effects of Intravenous Indomethacin on Reduction of Symptomatic Patent Ductus Arteriosus Cases and Decreasing the Need for Prolonged Mechanical Ventilation

**DOI:** 10.15171/jcvtr.2014.022

**Published:** 2014-12-30

**Authors:** Abdollah Jannatdoust, Mahmoud Samadi, Saadollah Yeganehdoust, Mohammad Heydarzadeh, Hossein Alikhah, Reza Piri, Mohammad Naghavi-Behzad

**Affiliations:** ^1^Children Health Research Center, Tabriz University of Medical Sciences, Tabriz, Iran; ^2^Students’ Research Committee, Tabriz University of Medical Sciences, Tabriz, Iran; ^3^Liver and Gastrointestinal Disease Research Center, Tabriz University of Medical Sciences, Tabriz, Iran; ^4^Medical Philosophy and History Research Center, Tabriz University of Medical Sciences, Tabriz, Iran

**Keywords:** Indomethacin, Patent Ductus Arteriosus, Hemorrhage, Mechanical Ventilation

## Abstract

***Introduction:*** We decided to investigate the effects of injecting Indomethacin on reducing complications of Patent Ductus Arteriosus (PDA) and the need for prolonged mechanical ventilation.

***Methods:*** During this randomized clinical trial, 70 premature infants with matched gestational age and birth weight were divided into case and control groups. In the study group, intravenous indomethacin started from the first 2-12 hours of birth. All patients were followed by echocardiography at the fourth day and skull ultrasound in the second week.

***Results:*** Symptomatic PDA rate was significantly higher in the control group (25.7% vs. 0%; P≤0.001). Incidence of grade 1-3 intraventricular hemorrhage was higher in the control group and the ratio of needed time for respiratory support in the control group to the case group was approximately 2.1.

***Conclusion:*** Intravenous Indomethacin reduced the number of PDA cases and incidence of grade 2 and 3 intraventricular hemorrhage, without any short term side effects.

## Introduction

The main mechanism of arterial duct closure has not understood yet, but increasing oxygen pressure and releasing vasoactive agents have important roles in duct’s closure process in after-birth step. Prostaglandins (PG) E1, E2, and I2 cause arterial ducts to remain open and prostaglandin synthesis inhibitors cause shrinkage of arterial ducts. Its shrinkage and closure starts from pulmonary artery side and continues to aorta in a cone shape.^1,2^

Spontaneous closure of Patent Ductus Arteriosus (PDA) could happen in the first year, but since it increases the risk of endocarditis, its closure is suggested to happen in 6-month age, but drug-induced or surgical closure is needed for infants who are very birth low weight (under 1000 g).^2-4^

PDA therapy consists of restricting fluid intake, administering prostaglandin inhibitors (such as indomethacin) and surgical closure.^4^ After birth, indomethacin causes shrinkage, ischemia and rearrangement in the structure of the arterial duct, duct closure and reduces the need for surgical intervention.^3,5^ So this motivated clinical researchers to use this drug after birth upon suspected initial symptoms.^5,6^

This study was aimed to investigate the effects of intravenous indomethacin on reducing complications of PDA and the need for prolonged ventilatory support.

## Materials and methods

In this randomized clinical trial which was conducted in neonatal intensive care unit of Alzahra Educational-Medical center (Tabriz, Iran), during June of 2010 till December of 2012, 70 premature infants with under 32 weeks age and birth weight of 800-1500 g were included in study.

Patients were excluded if they had any of the exclusion criteria. Exclusion criteria was consisted of:

1- Certain congenital abnormalities
2- Despite the severe asphyxia (Fifth minute apgar score < 7 or being initial pH less than 7.1)
3- Moderate thrombocytopenia (50,000/μL).
4- High serum creatinine (Cr) 1.8 mg/dl
5- The obvious bleeding (respiratory, digestive, urinary, skin or mucous)
6- Receiving indomethacin by mother.

Explaining the parents that there were not any reported side effects of prophylactic intravenous indomethacin in premature infants as well as its reported benefits, patients were included in study after the written consent was obtained.

Infants were randomly divided into case and control groups (35 in each group) using Rand list software. In the case group, intravenous indomethacin injection with 0.2 mg/kg dose started from first 2-12 hours and continued after 24 and 48 hours with 0.1 mg/kg dose. On the fourth day of birth, thoracic echocardiography was conducted for both groups and in the second week skull ultrasonography through fontanel was performed. Primary blood pH was examined in both groups in neonatal intensive care unit on admission time and. Cr and platelets were measured on the first and fourth days. Both groups were investigated for Intermittent Mandatory Ventilation (IMV), Continuous Nasal Positive Airway Pressure (NCPAP), High-Flow Nasal Canola (HFNC) or oxy-hood.

Statistical analysis was performed by SPSS-16 (SPSS Inc., Chicago, USA). Quantitative data were presented as mean ± standard deviation (SD), while qualitative data were demonstrated as frequency and percent (%). The collected data were studied using descriptive statistical methods, the mean difference test for independent groups, and Chi-squaretest or Fisher’s exact test. P value less than 0.05 was considered significant.

## Results

Each group included 18 girl (51.4%) and 17 boys (48.6%). The gestational age was 28.9±1.21 weeks in study group and 29.51±1.65 weeks in controls (P=0.8).

Groups were not statistically different in birth weight, Apgar score of the 1^st^ and 5^th^ minutes, endotracheal surfactant, time for receiving first dose of surfactant, serum Cr, platelet count in the 1^st^ and 4^th^ days and primary pH (P>0.05).

Eight infants (22.8%) from study group were under ventilator for 1.46±0.66 days, but in control group 10 infants (28.5%) underwent ventilation for 4.16±2.93 days. Also 31 infants (94.92%) from study group needed for NCPAP for 4.17±4.26 days and 31 infants (88.57%) in control group needed it for 2.03±1.27 days.

The difference of two groups in need for ventilation and continuous positive airway pressure (C-PAP) was 2.85 and 2.05 times, respectively. There was similar value in using nasal cannula with high frequency or oxy hood and significant difference was found in reduction of time needed for interventions. Prophylactic intravenous indomethacin caused significant differences in need for ventilation, C-PAP, and in using nasal cannula with high frequency or oxy hood. In other words this drug led to reduction of need for respiratory support in all mentioned variables of study group.

In study group, 21 infants (60.6%) had no other heart findings, 10 infants (28.4%) had atrial septal defect, 2 infants (5.7%) had mitral regurgitation and 2 infants (5.7%) had tricuspid regurgitation, but in control group, 25 infants (71.4%) had no other heart findings, 8 infants (22.9%) had atrial septal defect, 1 infant (2.9%) had ventricular septal defect, and 1 infant (2.9%) had mitral regurgitation.

Auditory symptoms in clinical examination were 10.9 times more prevalent in control group. Three infants (8.57%) were expired in control group while one infant (2.85%) in study group. Other complementary results are summarized in Table 1.

**Table 1:** Comparison of Intervention and Control groups among the study factors

	**Preterm birth** **(maternal preeclampsia)**	**Preterm birth(PPROM)**	**Preterm birth** **(multiple pregnancy)**	**PDA symptoms**			**Transfontanelle sonography**				**Pulmonary artery pressure**			**Pulmonary hemorrhage**	**Echocardiography**		
				**Bond pulse**	**Systolic murmur**	**Normal**	**IVH-G1**	**IVH-G2**	**IVH-G3**	**IVH-G4**	**Normal**	**Mild PH**	**Mod PH**		**No PDA**	**Small PDA**	**Large PDA**
**Intervention Group**				0	1	27	3	2	1	1	31	3	0	3	33	2	0
**N (%)**	37.9	18.0	11.5	0	2.9	79.4	8.8	5.7	2.9	2.9	91.2	8.8	0	8.5	94.1	5.9	0
**Control Group**				9	11	15	8	9	2	1	25	8	2	8	16	10	9
**N (%)**	40.6	18.8	9.4	25.7	31.4	42.9	22.9	25.7	5.7	2.9	71.4	22.9	5.7	22.9	45.7	28.6	25.7

## Discussion

This study showed that symptomatic PDA, Grade 1-3 IVH, and duration of respiratory support were significantly higher in the control group.

One of the important goals of prophylactic intravenous indomethacin is preventing PDA and reducing associated complications in infants with extremely low body weight (ELBW) by early and permanent closing of arterial ducts.^3^

Spontaneous arterial duct closure happens in less than 40% of infants under 26 weeks and in more than 70% of infants over 30 week^3^, and prophylactic indomethacin in the first 6 hours of birth have reduced symptomatic PDA in infants under 1000 g and also decreased IVH grade 3 & 4 cases.^7^ In our study, prevalence of PDA in control group was 9 times of that of study group.

Starting intravenous indomethacin in the first 12 hours in infants under 30 weeks and weight of 400-1250 have reduced PDA cases and the need for surgery.^8^ This was also obvious in our study.

Prophylactic intravenous indomethacin causes reduction in need for treatment of PDA complications.^9,10^ In our study there were not significant differences between two groups in severe IVH and NEC.

In a Cochrane review about giving prophylactic intravenous indomethacin for reducing mortality and disability it was mentioned that in infants under 1500 g it reduces symptomatic PDA cases and reduces IVH grade 3 & 4. It also has been mentioned that giving intravenous indomethacin has not significant effect on short term and long term mortality and also on incidence of NEC and long term nervous evolution is yet under study. About increase probability of Retinopathy of Prematurity (ROP) in infants who had received indomethacin, there was no statistical significant difference in ROP grades. ROP was not investigated in our study.^5,11^

Our study showed that giving prophylactic intravenous indomethacin to preterm infants who are equal and under 32 weeks leads to 2.8 times reduction in the number of ventilator needed days, and 2 times reduction in need for NCPAP in study group. Also it showed that giving indomethacin causes 5 times reduction in prevalence of small PDA and no large PDA case was found in study group.

Comparing pulmonary arterial hypertension by Doppler echocardiography, mild PH cases are 2.6 times more prevalent and bond pulse and moderate pH were not found in study group.

## Conclusion

Administering prophylactic intravenous indomethacin for under 32 weeks preterm infants causes a reduction in symptomatic PDA cases, need for mechanical ventilation, NCPAP and oxygen, reduction of IVH grade 1-3 and reduction in short term mortality, but no difference was found in IVH grade 4 amount between two groups.

## Acknowledgments

This research was supported by a grant from Children Health Research Center of Tabriz University of Medical Sciences, Tabriz, Iran.

## Ethical issues

This study was approved by the ethics committee of Tabriz University of Medical Sciences (Registration code: 5/4/2573). This study was also registered at Iranian Registry of Clinical Trials by code of IRCT201107117010N1 (http://www.irct.ir)

## Competing interests

Authors declare no conflict of interests in this study.
